# Lessons from the first *e*cancer symposium on angiogenesis in gastric cancer

**DOI:** 10.3332/ecancer.2015.553

**Published:** 2015-07-21

**Authors:** Audrey Nailor, Elisabetta Dejana, Andrew R Reynolds, Shonit Punwani, Giuseppe Curigliano, Francesco Bertolini, Manish Shah, Romano Danesi, Robert Kerbel, Gordon McVie

**Affiliations:** 1 *e*cancer, 154 Cheltenham Road, Bristol BS6 5RL, UK; 2 Vascular Biology Programme, IFOM, FIRC Institute of Molecular Oncology Foundation, Via Adamello 16, 20139 Milan, Italy; 3 Tumour Biology Team, The Breakthrough Breast Cancer Research Centre, The Institute of Cancer Research, 237 Fulham Road, London SW3 6JB, UK; 4 University College London, Gower Street, London WC1E 6BT, UK; 5 Division of Experimental Therapeutics, European Institute of Oncology, Via Ripamonti 435, 20141 Milan, Italy; 6 Laboratory of Haematology-Oncology, European Institute of Oncology, Milan 20141, Italy; 7 Weill Cornell Medical College, New York 10065, USA; 8 University of Pisa, Pisa 56126, Italy; 9 Department of Medical Biophysics, University of Toronto, ON M4N 3M5, Toronto, Canada; 10 European Institute of Oncology, Milan 20146, Italy; University of Milan, Italy; University of Glasgow, G12 8QQ, United Kingdom; University of Wales, Cardiff, South Glam CF10 3NS, United Kingdom; Founding editor of *ecancer.org*

**Keywords:** angiogenesis, gastric cancer, cancer development, cancer education, cancer outreach

## Abstract

In March 2015, *e*cancer hosted a symposium at the European Institute of Oncology in Milan, Italy on the topic of angiogenesis in gastric cancer.

During this meeting, leaders in the field focused on the latest research on the topic of angiogenesis in gastric cancer, delivering lectures combined with interactive question and answer (Q & A) sessions and a roundtable discussion with the meeting’s chairs. Topics covered included biomarkers, imaging, and the current state of antiangiogenic drugs in gastric cancer.

This report will provide an understanding of the relevance of angiogenesis in gastric cancer research, and clinical experiences from diverse perspectives.

## Introduction

In March 2015, *e*cancer hosted a symposium at the European Institute of Oncology in Milan, Italy on the topic of angiogenesis in gastric cancer. The one-day workshop was delivered by an international faculty, hand-picked by Prof Gordon McVie of *e*cancer, Bristol, UK and Prof Francesco Bertolini of the European Institute of Oncology. The event was also chaired by Prof Robert Kerbel of the University of Toronto, Canada.

During this meeting, leaders in the field focused on the latest research on the topic of angiogenesis in gastric cancer, delivering lectures combined with interactive Q & A sessions, and a roundtable discussion with the meetings’ chairs. Topics covered included biomarkers, imaging, and the current state of antiangiogenic drugs in gastric cancer.

The event was free of charge to oncologists living in Italy, and it has been released on *e*cancer’s website as a free webcast and as a e-learning module.

This report will provide an understanding of the relevance of angiogenesis in gastric cancer research, and the clinical experiences from diverse perspectives.

## Background

Angiogenesis is the process by which new blood vessels grow from existing ones; vasculogenesis refers to the *de novo* growth of blood vessels. Angiogenesis is a topic of significant interest in several research areas, including evolution and development, cardiovascular health, and the study of diabetes. Many of these research fields are interested in stimulating angiogenesis and vasculogenesis, particularly to repair the damaged tissue.

In cancer research, the goals are reversed: how can angiogenesis be slowed or halted? Angiogenesis plays an important role in tumour growth and development. Tumours like other tissue require a supply of blood and as tumours grow, they co-opt the host’s blood supply, encouraging blood vessels to sprout additional lines. The mechanisms by which cancerous tissues vascularise are of key interest when studying and treating cancer.

Thus, antiangiogenic drugs–which prevent the growth and development of these tumour-feeding vessels–are an emerging field of research. The question now is how can they be applied in the challenging field of gastric cancer?

## Presentations

The one-day workshop featured eight presentations from international researchers. The initial presentations provided background into angiogenesis, its biology and mechanisms. It was aimed at students and healthcare professionals who may not have included these processes in their studies or studied them in great detail.

Dr Elisabetta Dejana of the University of Milan, Milan, Italy laid the groundwork for the meeting with her presentation on the basic biology of angiogenesis and vasculogenesis [1]. She provided key definitions of important terms, such as the famed vascular endothelial growth factor (VEGF). VEGF stimulates angiogenesis and is secreted by cancer cells and is thus a key target for antiangiogenic drugs.

Dr Andrew R Reynolds, of The Institute of Cancer Research, London, UK built on this foundation with his presentation on the pathophysiology of the vascular supply in cancer [2]. He noted that existing antiangiogenic drugs target the VEGF signalling pathway by different mechanisms, including bevacizumab (binds to the VEGF ligand), ramucirumab (binds to the VEGFR2 receptor), and sunitinib (inhibits VEGF receptor kinase activity). He reported that VEGF-targeted drugs had made an impact in the treatment of some cancers, but not in others, and that a key challenge is understanding the mechanism of resistance to antiangiogenic therapy. Dr Reynolds said that the mechanisms of resistance are diverse and poorly understood. He highlighted redundancy of pro-angiogenic growth factor signalling and vessel co-option as being two important forms of resistance to antiangiogenic therapy.

Can these processes be visually observed happening within the patient? Dr Shonit Punwani, of the University College London, London, UK gave a presentation on the use of magnetic resonance imaging (MRI) to observe vascularity in cancer. A key take-home message in Dr Punwani’s lecture was that there is currently low awareness of this application in the field, but that MRI is a feasible way to assess vascularity [3]. Dynamic contrast enhanced (DCE)-MRI is the most commonly employed technique; he believes that vascularity should be used in conjunction with other information about the tumour as part of a multi-parametric evaluation.

How are antiangiogenic treatments faring in the clinical setting? Dr Giuseppe Curigliano of European Institute of Oncology, Milan discussed the clinical use of antiangiogenic drugs and their results. Dr Curigliano updated the audience on recent and ongoing clinical trials of relevance. Particularly positive were the recently published RAINBOW and REGARD trials, which demonstrate that ramucirumab is an ‘effective’ drug for the second-line treatment of patients with metastatic or locally advanced unresectable gastric cancer [4]. He also addressed the potential future role of immuno-checkpoint inhibitors in the treatment of gastric cancer, also in combination with metronomic schedules of chemotherapy.

Clearly, biomarkers will provide key information to researchers. Prof Francesco Bertolini of the European Institute of Oncology, Milan lectured on the applications of angiogenesis biomarkers, including personalised cancer medicine [5]. Summarising the latest research, he suggested that angiogenesis biomarkers—such as gene polymorphisms, increased VEGF levels, and circulating cells—have the potential to predict treatment response in gastric cancer.

Dr Manish Shah of Weill Cornell Medical College, New York, USA provided a North American perspective on the present positioning of antiangiogenesis drugs within the gastric cancer clinical landscape [6], while Prof Romano Danesi of the University of Pisa, Pisa, Italy provided an European counterpoint view [7].

Dr Shah reminded the audience that gastric cancer is not a single entity, but a linked network of related diseases. He noted the current state of research into targeting gastric cancer; angiogenesis is an increasing area. Shah himself is a leader of the AVAGAST trial, examining bevacizumab as first-line antiangiogenic treatment of gastric cancer, though here he also touched on the RAINBOW and REGARD trials. He followed on Bertolini’s observations by noting the impact of biomarker profiles on the outcome of treatment with bevacizumab.

Prof Danesi then provided a European perspective on the clinical use of antiangiogenic therapies in gastric cancer, where drugs are subject to different laws. The European Commission granted a marketing authorisation for ramucirumab in December 2014, although some restrictions are still in place. European healthcare professionals and policymakers are cautiously optimistic, awarding ramucirumab a ‘black triangle’ label indicating that it is being carefully monitored. Prescribers are asked to report any suspected adverse reactions. Prof Danesi is optimistic that emerging drugs, such as lapatinib, may widen the European treatment landscape.

Throughout the event, speakers noting the interaction between chemotherapy and antiangiogenics referred to the upcoming presentation by Prof Robert Kerbel, of the Sunnybrook Research Institute and the University of Toronto, Canada [8]. Prof Kerbel tied off these loose ends by finishing the workshop with his anticipated talk on the combination of chemotherapy and antiangiogenic therapy, and the factors which should inform treatment decisions. He explained that conventional wisdom has found that antiangiogenic antibody treatments bevacizumab and ramucirumab interact with chemotherapy by improving its efficacy, and outlined some hypotheses for this mechanism. But what if the opposite of conventional wisdom is true—could it be that chemotherapy improves the efficacy of the antiangiogenic drug as well as the antiangiogenic drug improving the impact of the chemotherapy? Prof Kerbel summarized the results of several preclinical studies showing that antiangiogenic drug monotherapy can sometimes increase local tumor invasion and/or distant metastasis—perhaps by increasing hypoxia. However, this potentially undesirable effect of the antiangiogenic drug can be prevented or blocked by concurrent chemotherapy. Thus, the nature of the chemotherapy treatment (which he referred to as the ‘partner’ or ‘backbone’ treatment) is both highly significant and complex and emphasizes the need to select the chemotherapy partner carefully, based on appropriate mechanism-based studies.

The workshop ended with a Q & A session, in which the audience quizzed the three chairs–Profs McVie, Bertolini, and Kerbel–on the content of the lectures as well as asking for opinions and advice.

## Conclusions

The goal of the ‘Angiogenesis: Latest Developments in Gastric Cancer’ workshop was to enable oncologists to understand the latest advances and exploitation of angiogenesis in gastric cancers.

The audience were provided with the latest emerging evidence in the optimal integration of antiangiogenic therapy into the treatment plan for gastric cancer patients, to be applied in the future to clinical practice.

The rapidly evolving field of antiangiogenic therapies in gastric cancer challenges clinicians to stay abreast of the latest developments. The background information required to grasp the processes of angiogenesis might not have been consistently distributed across the educational backgrounds of clinicians.

Continuing medical education is a key bridge for this potential knowledge gap. This selection of talks was intended to be such a bridge, informing, and educating cancer professionals across a broad spectrum of career stages—from medical students to practicing oncologists to policymakers.

It is recommended that the interested reader access the free online webcast to deepen and broaden their understanding of the talks covered in this report.

## References

[1] Dejana Elisabetta (2015). Basic biology of angiogenesis and vasculogenesis. webcast ecancer.

[2] Reynolds Andrew (2015). Pathophysiology of vascular supply in cancer. webcast ecancer.

[3] Punwani Shonit (2015). Using MRI to assess vascularity in cancer. webcast ecancer.

[4] Curigliano Giuseppe (2015). Candidate antibodies and small molecules in early clinical trials in gastric cancer. webcast ecancer.

[5] Bertolini Francesco (2015). Biomarkers of angiogenesis including imaging to assist patient choice of therapy and subsequent management. webcast ecancer.

[6] Shah MA Clinical experience in gastric cancer and present positioning of anti-angiogenesis drugs (North American perspective). webcast ecancer.

[7] Danesi Romano (2015). Clinical experience in gastric cancer and present positioning of anti-angiogenesis drugs (European perspective). webcast ecancer.

[8] Kerbel Robert (2015). Choice of chemotherapy to accompany antiangiogenesis drugs. webcast ecancer.

